# Identification and Integrated Analysis of MicroRNA and mRNA Expression Profiles During Agonistic Behavior in Chinese Mitten Crab (*Eriocheir sinensis*) Using a Deep Sequencing Approach

**DOI:** 10.3389/fgene.2020.00321

**Published:** 2020-04-23

**Authors:** Yangyang Pang, Long He, Yameng Song, Xiaozhe Song, Jiahuan Lv, Yongxu Cheng, Xiaozhen Yang

**Affiliations:** National Demonstration Center for Experimental Fisheries Science Education, Key Laboratory of Freshwater Aquatic Genetic Resources, Ministry of Agriculture, Engineering Research Center of Aquaculture, Shanghai Ocean University, Shanghai, China

**Keywords:** *Eriocheir sinensis*, agonistic behavior, microRNA, mRNA, neurogenesis, energy metabolism

## Abstract

As a commercially important species, the Chinese mitten crab (*Eriocheir sinensis*) has been cultured for a long time in China. Agonistic behavior often causes limb disability and requires much energy, which is harmful to the growth and survival of crabs. In this paper, we divided crabs into a control group (control, no treatment) and an experimental group (fight, agonistic behavior after 1 h) and then collected the thoracic ganglia (TG) to extract RNA. Subsequently, we first used a deep sequencing approach to examine the transcripts of microRNAs (miRNAs) and messenger RNAs (mRNAs) in *E. sinensis* displaying agonistic behavior. According to the results, we found 29 significant differentially expressed miRNAs (DEMs) and 116 significant differentially expressed unigenes (DEGs). The DEMs esi-miR-199a-5p, esi-let-7d, esi-miR-200a, and esi-miR-200b might participate in the regulation of agonistic behavior by mediating neuroregulation and energy metabolism. Focusing on the transcripts of the mRNAs, the renin–angiotensin system (RAS) Kyoto Encyclopedia of Genes and Genomes (KEGG) pathway might be involved in the regulation of agonistic behavior through glucose metabolism as this pathway was significantly enriched with DEGs. Besides, an integrated analysis of the miRNA and mRNA profiles revealed that the retinoid X receptor (RXR) was also involved in visual signal transduction, which was important for agonistic behavior. In addition, four vital agonistic behavior-related metabolic pathways, including the cAMP signaling, MAPK, protein digestion and absorption, and fatty acid metabolism pathways, were significantly enriched with the predicted target unigenes. In conclusion, the findings of this study might provide important insight enhancing our understanding of the underlying molecular mechanisms of agonistic behavior in *E. sinensis*.

## Introduction

Chinese mitten crab (*Eriocheir sinensis*) has been loved by Chinese people and cultured in many areas of China for a long time (Cheng et al., 2018). Agonistic behavior is a common phenomenon in crustaceans and usually decreases the crabs’ integrity, survival, and growth (Laranja et al., 2010; Harlıoğlu et al., 2013; Romano and Zeng, 2016). In our previous studies, we reported the agonistic behavior of *E. sinensis* and further investigated the regulation of serotonin (5-HT) and dopamine (DA) in agonistic behavior through the cAMP signaling pathway (Pang et al., 2019a; Yang et al., 2019). First, we had divided the agonistic behavior in *E. sinensis* into three stages: approach, contact, and fight; and then, we found a sign in the concentration of 5-HT, DA, cAMP, and PKA after agonistic behavior (Yang et al., 2019). Subsequently, we injected the 5-HT, DA, or cAMP to crabs and observed the effect of agonistic behavior after injection. The results showed that 5-HT and DA could regulate the behavior through the cAMP–PKA signaling pathway (Pang et al., 2019a). However, the mechanism underlying agonistic behavior in *E. sinensis* is unclear, and research investigating this topic is lacking. An understanding of the regulatory mechanisms underlying the switching of agonistic behavior is essential for elucidating the behavioral plasticity of animals and ultimately decreasing the occurrence of agonistic behavior.

MicroRNAs (miRNAs) constitute a class of non-coding RNAs with a length of 18–22 nt that can regulate gene expression and usually play an important role in many biological processes (Bartel, 2004; Wang L. et al., 2018). According to reports, miRNAs could mediate neurogenesis at many key steps in vertebrate and invertebrate (Li and Jin, 2010) and regulate animal behaviors through nervous systems (Lane et al., 2018). For example, miR-375 and miR-200b could regulate attraction/aversion behavior via the dopaminergic and GABAergic systems in amphibians (Dulcis et al., 2017). Another study showed that the overexpression of let-7d perfected the anxiolytic and depressant-like behaviors by targeting the DA D3 receptor (Bahi and Dreyer, 2018). Researchers have also found that miR-96, which could inhibit the 5-HT1B receptor, affected aggressive behavior in mice (Jensen et al., 2009). Although many reports described the effects of miRNAs on animal behaviors, studies investigating the effects of miRNAs on aggressive behavior in crustaceans were lacking. Combined with our previous researches, 5-HT and DA as two neurotransmitters played key roles in nervous systems and regulated the agonistic behavior in *E. sinensis*. So to research the relationship between miRNAs and nervous systems would undoubtedly be of great help to understand the regulatory mechanisms of agonistic behavior. In addition, agonistic behavior usually was accompanied by energy metabolism (Briffa and Elwood, 2002). We also found that the glucose level has a significant upregulation after the fight in *E. sinensis* (Yang et al., 2019). There were reports that miRNAs could regulate the energy metabolism in animals; for example, miR-199a-5p could participate in the regulation of glucose metabolism (Wu et al., 2015; Yan et al., 2017; Esteves et al., 2018). Therefore, the relationship between miRNAs and energy metabolism was valued in research on agonistic behavior.

The central nervous system (CNS) plays a major role in regulating several behaviors in some invertebrates, including agonistic behavior (Momohara et al., 2018), reproduction behavior (Thongbuakaew et al., 2019), and phototactic behavior (Rivetti et al., 2018). Besides, the thoracic ganglia (TG) is an important nerve tissue that participates in the formation of the CNS in crustaceans (Mulloney and Smarandache-Wellmann, 2012). The TG is among the many places where many neurotransmitters transfer biological information, such as DA, 5-HT, octopamine (OA), and tyramine (TA) (Vomel and Wegener, 2008). In our previous studies, we had done a lot of research on TG, including *in vitro* culture and RT-qPCR (Pang et al., 2019a). Based on the measurements of the TG, we found that 5-HT and DA could regulate the agonistic behavior of *E. sinensis* through the TG (Pang et al., 2019a; Yang et al., 2019). Therefore, investigating the TG was suitable for analyzing the mechanism underlying agonistic behavior in *E. sinensis*.

In this study, we obtained the transcriptomes of miRNAs and mRNAs after agonistic behavior by using Illumina HiSeq^TM^ 2000 technology. Also, we analyzed the functions of the significant differentially expressed miRNAs (DEMs) and mRNAs in the TG and performed an integrated analysis of their relationships. We aimed to reveal the underlying molecular mechanisms involved in the regulation of agonistic behavior in *E. sinensis*.

## Materials and Methods

### Animals and Sampling

Male Chinese mitten crabs (*E. sinensis*) with a body weight of 25.56 ± 4.73 g were obtained from the Shuxin crab base in Chongming Island, Shanghai (China). The crabs were maintained in separate opaque tanks of 29.0:18.0:19.5 cm (length:width:height) for at least 7 days under single-rearing conditions before the behavioral experiments, and the tanks were filled with thoroughly aerated freshwater to a depth of 12 cm by a circulating system. The intact crabs were reared individually to avoid social contact. A basal diet was used to feed the crabs once daily from 18:30 to 19:00.

The crabs were divided into a control group (control) and an experimental group (fight). In the experimental group, two crabs with a weight difference of 1–4% were paired in a new tank of 20.0:15.5:19.5 cm (length:width:height). The tank was filled with 10 cm of water and divided into equal halves by an opaque partition. Before each pairing, each pair of crabs was placed on either side of the partition. After 10 min, the partition board was removed, and the agonistic behavior of the crabs was observed for 1 h using a high-definition camera (H.264 DVR) based on our previous research (Yang et al., 2019). We ensured that agonistic behavior occurred within 1 h, and then, the TG were collected for deep sequencing. In the control group, the crabs were placed on either side of the partition for 70 min, and then, their TG were harvested.

### RNA Extraction and Quality Check

The total RNA was extracted using a TRIzol reagent (Sangon Biotech Co., Ltd.) according to the manufacturer’s instructions. After RNA extraction, every two crabs which were collected from one pair were mixed as one sample to subsequent detection. Qubit 2.0 (Invitrogen, United States) was used to determine the concentration and purity of the total RNA. The integrity of the RNA and genome contamination were examined by agarose gel electrophoresis.

### Small RNA Library Construction and Illumina Sequencing

Based on the above results, three samples from the fight group (named F1, F2, and F3) and another three samples from the control group (named C1, C2, and C3) were used to construct six small RNA libraries. In each library, a 6 μl mixture contained approximately 1 μg or more total RNA, and 1 μL 3′ adapter and DEPC H_2_O were used to prepare the RNA mix; then, the mixture was incubated at 72°C for 2 min. Subsequently, 1.8 μl 10 × T4 RNA ligase buffer, 3.5 μl 25 mM MgCl_2_, 2.2 μl PEG8000 (50%), 0.5 μl RNase inhibitor, and 1 μl ligase 2 were added to the RNA mix. Then, 15 μl of the mixture was incubated at 22°C for 1 h, 1 μl RT primer was added to the above mixture, and the reaction conditions used were as follows: 75°C for 5 min; 37°C for 30 min, and 25°C for 15 min. To ligate with the 5′ adapter, 16 μl of the above hybrid production was opened with a secondary structure at 70°C for 2 min, and then, 1 μl ATP (10 mM), 1 μl 5′ adapter, and 1 μl ligase 1 were added. The obtained reaction solution was incubated for 1 h at 20°C. Then, the adaptor-ligated small RNAs were reverse transcribed to create the complementary DNA (cDNA) constructs. Subsequently, the cDNA constructs were amplified by PCR using an Illumina small RNA primer set and Phusion polymerase under the following conditions: 95°C for 5 min; 15 cycles of 94°C for 10 s, 55°C for 10 s, and 72°C for 15 s; 72°C for 5 min; and 4°C for ∞. The productions of PCR were detected by 12% polyacrylamide (PAGE). The reads were obtained with a single end sequencing pattern at Sangon Biotech Co., Ltd. (Shanghai, China) using Illumina HiSeq^TM^ 2000.

### MRNA Library Construction and Sequencing

Simultaneously, we also selected three samples from the fight group (named F4, F5, and F6) and another three samples from the control group (named C4, C5, and C6) to construct six mRNA libraries. The samples used for the mRNA transcriptome analysis were prepared according to a VAHTS^TM^ mRNA-seq V2 Library Prep Kit for Illumina^®^ (Vazyme Biotech Co., Ltd., Nanjing, China). Poly-T oligo-linked magnetic beads were used to purify the PolyA mRNA from the total RNA, and the intact mRNA was broken into fragments with a bead washing buffer and a metal bath. The aforementioned mRNAs were used as templates to synthesize the first-strand cDNA. Then, the second-strand cDNA was synthesized using the second-strand buffer and second-strand enzyme mix. Subsequently, the cDNA was end-repaired, a base was added to the 3′ end, and the cDNA was amplified by PCR. Finally, the constructed cDNA libraries were sequenced with Illumina HiSeq^TM^ 2000.

### Analysis of miRNA and mRNA Sequence Data

The adapter sequences (sequence: TGGAATTCTCGGGTGCC AAGGAACTC) were removed from the raw data using the cutadapt software. In addition, the low-quality reads (a base quality less than Q20, the average quality of four consecutive bases less than Q20 and reads shorter than 17 nucleotides) were filtered using trimmomatic (Bolger et al., 2014). Finally, the clean reads of 17–35 nt were used in the subsequent analysis.

The clean reads may contain other small RNAs (rRNAs, sRNAs, snRNAs, and snoRNAs), which were compared using the rfam (Nawrocki et al., 2015) database and blastn. The conditions of the alignment were as follows: gapopen = 0, evalue < 0.01, and mismatch ≤ 1.

For the animal miRNAs, miranda (Betel et al., 2008) was used to predict the target genes. The predicted miRNA targets were based on a threshold value for the parameters as follows: S ≥ 150, ΔG ≤ −30 kcal/mol. The target genes were predicted based on the *E. sinensis* mRNA transcriptome sequence obtained in this study as a reference genome.

Regarding the mRNA sequencing, raw reads from Illumina HiSeq^TM^ 2000 may contain sequencing primers and low-quality sequences, which can affect the analytical quality. Therefore, the raw reads were cleaned through the following three steps: (a) linger sequences were discarded; (b) *Q* < 20 (*Q* = −10 log_10_E) bases were removed; and (c) read lengths shorter than 25 bp were discarded. Then, the clean reads were used for the *de novo* assembly using Trinity. Regarding the annotation, the assembled final unigenes with screening condition were annotated using the NCBI nucleotide sequence (NT) and NCBI protein nonredundant (Nr) databases. The unigenes were also classified using the Gene Ontology (GO) and Kyoto Encyclopedia of Genes and Genomes (KEGG) databases.

### Differential Expression Analysis of miRNAs and mRNAs

The expression levels of the miRNAs were determined by using RPM (normalized to determine the expression in transcripts per million). To identify the significant DEMs between two groups, the read counts of the samples were analyzed as input data using edgeR (Xiaobei et al., 2014). The screening conditions were as follows: *p*-value < 0.05 and | logFoldChange| > 1. The expression levels of the genes were determined by using the TPM (transcripts per million) method. An analysis of the differential expression levels across the samples was performed using DEGseq, and the significant differentially expressed genes (DEGs) were identified (*q*-value < 0.05, | FoldChange| > 2). Then, the significantly enriched terms were obtained by mapping the DEGs to the corresponding KEGG pathways.

### Quantitative Real-Time PCR-Verified miRNAs

Five DEMs were randomly chosen for a quantitative real-time PCR (RT-qPCR) analysis to validate the accuracy of the RNA-seq results. The total RNA (500 ng), which was returned after sequencing (three samples per group), was used as a template to synthesize first-strand cDNA with a miRNA 1st Strand cDNA Synthesis Kit (by stem loop: 5′-GTCGTATCCAGTGCAGGGTCCGAGGTA TTCGCACTGGA TACGAC-3′, Vazyme Biotech) according to the manufacturer’s instructions (Zhang et al., 2018). Specific primers were designed based on the DEMs with a miRNA design software (Vazyme designed) (Table 1).

**TABLE 1:** Primers selected for evaluating the miRNA expression levels with RT-qPCR.

**Primer name**	**Primer sequence (5′–3′)**
esi-miR-194-5p	CGCGTGTAACAGCAACTCCAT
esi-miR-192	GCGCTGACCTATGAATTGACAG
esi-let-7b-3p	GCGCGCTATACAACCTACTGC
esi-lig-miR-750	GCGCGCAGATCTAACTCTTCC
esi-miR-146b-5p	CGCGTGAGAACTGAATTCCA
snRNA	CGTTGGAACGATACAGAGAAGAT

The RT-qPCR reaction system contained 5 μl of 2 × miRNA Universal SYBR qPCR Master (Vazyme Biotech), 1 μl of diluted first-strand cDNA, 3.6 μl of PCR-grade water, 0.2 μl of specific primers, and 0.2 μl of mQ Primer R (5′-AGTGCAGGGTCCGAGGTA TT-3′) (Zhang et al., 2018). The mixtures were run under the following conditions: 95°C for 5 min; 40 cycles of 95°C for 10 s and 60°C for 30 s; and 95°C for 15 s, 60°C for 1 min, and 95°C for 15 s on an ABI 7500 Real-Time PCR System (Life Technology, United States). We used snRNA as an internal control (Liu et al., 2019). The DEM expression levels were calculated using the 2^–ΔΔCt^ method (Pang et al., 2019b).

## Statistical Analysis

The data are expressed as the mean ± SD, and a *T*-test analysis of variance was used to compare two groups. A *P*-value < 0.05 indicated a statistically significant difference.

## Results

### Overview of Small RNA Sequences in *E. sinensis*

**FIGURE 1:**
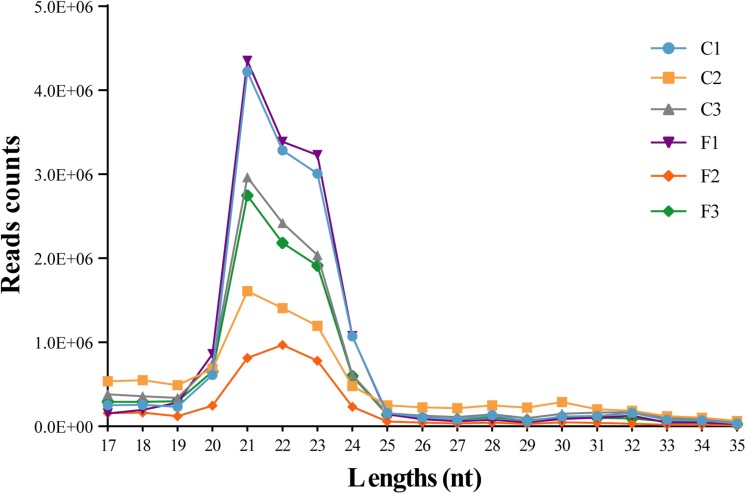
Length distribution of the clean reads from six siRNA libraries. The nucleotide length of the siRNAs is shown on the *X*-axis, and the counts of the different nucleotide lengths are shown on the *Y*-axis.

To explore the role of miRNAs in the regulation of agonistic behavior in *E. sinensis*, we obtained 34,367,222 clean reads from the control group and 28,150,606 clean reads from the fight group using Illumina HiSeq^TM^ 2000 (Supplementary Table S1). The small RNA sequences with lengths of 17–35 nt were the most enriched in 21–23 nt (Figure 1). As shown in Supplementary Table S2, the clean reads in the six small RNA libraries were composed of rRNAs, tRNAs, snRNAs, snoRNAs, and miRNAs.

### Identification of Mature miRNAs

The miRNA clean reads were blasted against the mature sequences of known miRNAs included in the miRbase (Kozomara and Griffiths-Jones, 2014), which revealed a total of 893 mature miRNAs in *E. sinensis*, and the number of mature miRNAs in each library is shown in Supplementary Table S2. When we summarized the conserved miRNAs, 64 miRNAs were specific to the fight group. While 129 miRNAs did not belong to any family, all remaining miRNAs belonged to 117 known families. The top five miRNA families included miR-10 (69 miRNAs), let-7 (48 miRNAs), miR-9 (30 miRNAs), miR-8 (26 miRNAs), and miR-25 (24 miRNAs).

Most miRNAs had a relatively low expression level, and the RPM of some miRNAs was even below 100 (Wang L. et al., 2018). In this study, eight mature miRNAs, namely, esi-miR-100-5p, esi-miR-263b, esi-let-7-5p, esi-miR-184-3p, esi-miR-100a-5p, esi-miR-276a-3p, esi-miR-7a, and esi-miR-83, had an expression level greater than 10,000 RPM in each library.

### DEM Analysis and RT-qPCR Verification

The levels of the DEMs between the control and fight groups were compared according to RPM values ≥ 5 as determined by a linkage hierarchical cluster analysis. We obtained 29 DEMs, namely, 8 upregulated and 21 downregulated DEMs, compared to the control group, indicating that these miRNAs might be involved in agonistic behavior-specific regulation (Figure 3).

**FIGURE 2:**
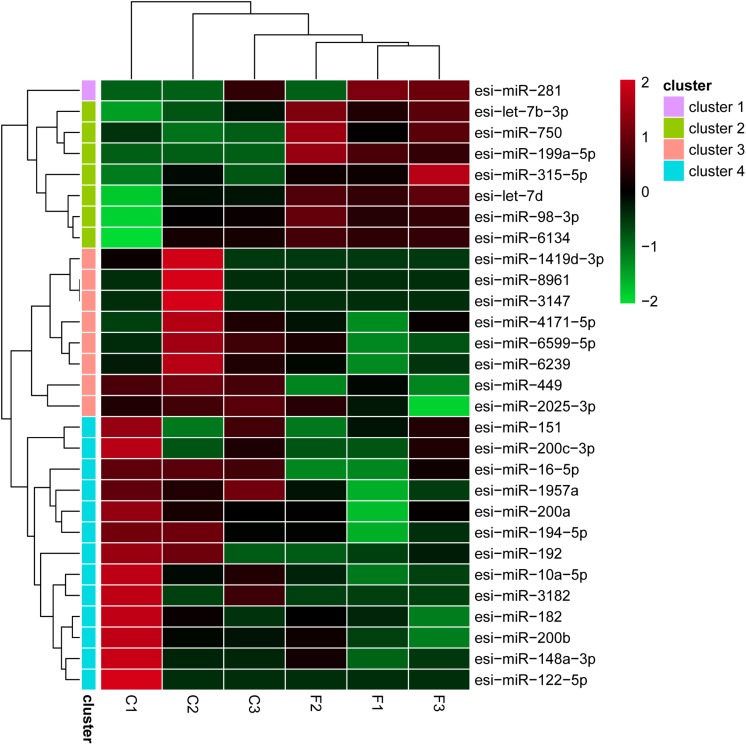
Differential expression of 29 miRNAs using a linkage hierarchical cluster analysis. The color indicates the log_2_-fold change from high (red) to low (green) as indicated by the color scale. The names of the miRNAs and the cluster to which they belong are shown on the right side.

Five DEMs were selected to verify the RNA-Seq results. The results of the RT-qPCR analysis confirmed that esi-miR-194-5p and esi-miR-192 were significantly downregulated in the fight group (Figure 2). Additionally, esi-let-7b-3p and esi-miR-750 were significantly upregulated in the fight group. We also found that esi-miR-146b-5p does not exhibit a significant difference in expression between the control and fight groups. Our results confirmed the data from the RNA-Seq.

### *De novo* Assembly and Unigene Functional Annotation

From RNA-Seq, we obtained 145,309,464 raw reads from the fight group and 142,210,006 raw reads from the control group. The clean reads were selected by excluding reads that did not conform to the requirements. Thus, there were 136,737,066 and 139,889,948 clean reads in the fight and control groups, respectively (Supplementary Table S1), and these reads were used for *de novo* assembly. Then, 217,281 unigenes were obtained from six mRNA libraries. In total, 18,950 unigenes had a length greater than 1,000 bp, and 53,333 unigenes were greater than 500 bp in length (Figure 4).

**FIGURE 3:**
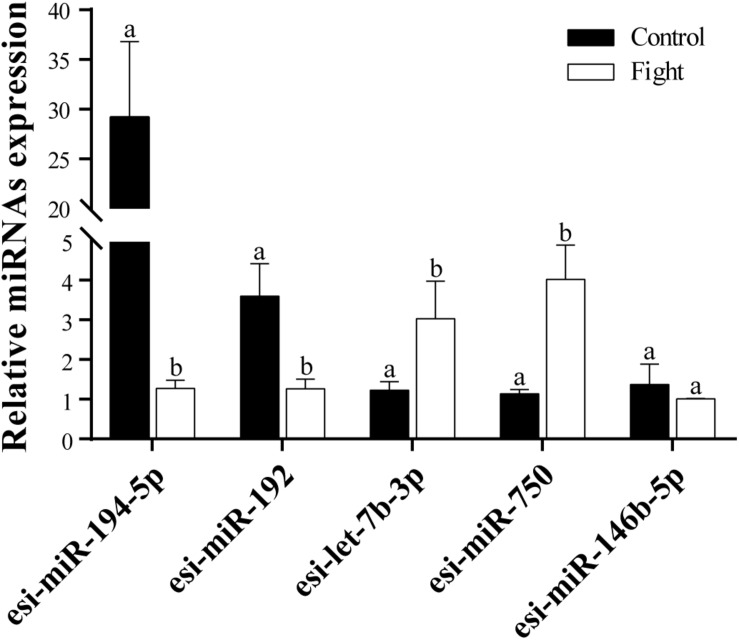
RT-qPCR analysis of five selected DEMs. Relative expression levels of tch-miR-194-5p, efu-miR-192, chi-Let-7b-3p, lgi-miR-750, and gga-miR-146b-5p are shown. The different lowercase letters above the bars indicates significant differences (*P* < 0.05) between the control and fight groups.

**FIGURE 4:**
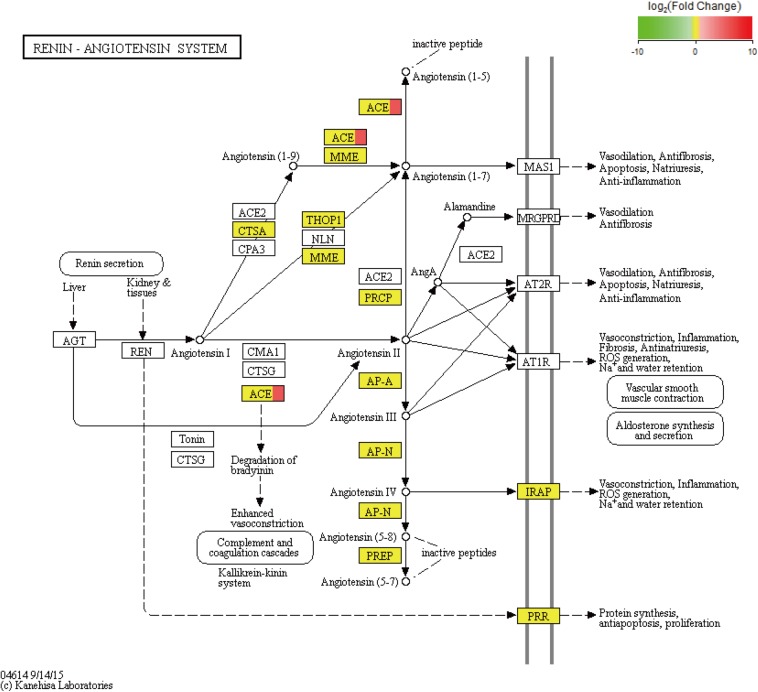
KEGG pathway of the renin–angiotensin system. In addition, the red color indicates upregulated genes, and the yellow color indicates genes with no significant differences in expression. ACE is angiotensin converting enzyme.

Besides, the unigenes were used for further functional analysis. In total, 30,458 unigenes were annotated by the GO database and assigned to the following three groups: molecular function, biological process, and cellular component; these groups were further divided into 57 categories. Most unigenes were concentrated in the binding, cellular process, and cell categories. The signaling pathways of 8,447 unigenes were analyzed using the KEGG KAAS online pathway analysis tool^1^. In addition, all unigenes were mapped to the following five processes: organismal systems (25.64%), metabolism (25.13%), genetic information (16.16%), cellular processes (15.90%), and environmental information processing (15.17%). Most unigenes were associated with signal transduction, translation, transport, and catabolism; carbohydrate metabolism; and endocrine system pathways.

### DEG Analysis

**FIGURE 5:**
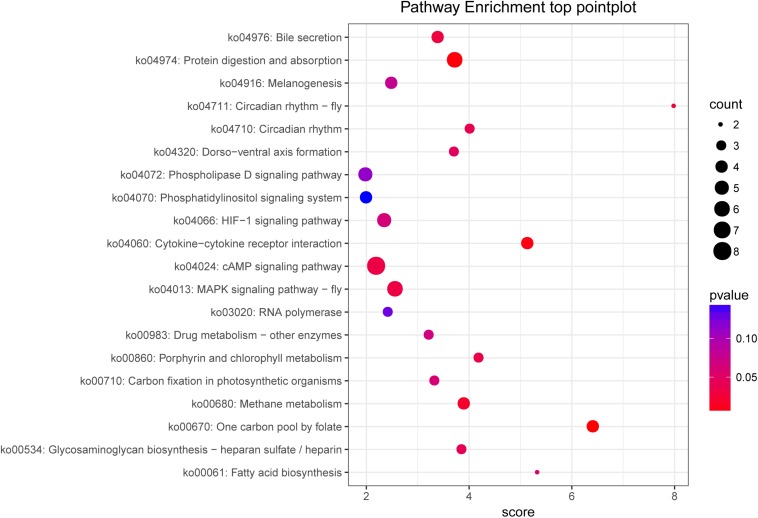
KEGG pathway enrichment of target genes between the control and fight groups. The abscissa represents the pathway enrichment grade. The *P*-value indicates the significance of the enrichment grade, and the circle size indicates the counts of the target genes.

We performed an analysis to detect significant differences in expression levels across the samples and found 101 upregulated significant DEGs and 15 significant downregulated DEGs between the fight and control groups. Furthermore, significant DEGs were assigned to KEGG pathways. The significantly enriched pathways (*q*-value < 0.05) included the renin–angiotensin system (RAS) (Figure 5), NOD-like receptor signaling pathway, cytosolic DNA-sensing pathway, renin secretion, and ribosome biogenesis in eukaryotes.

### MiRNA Target Gene Prediction and Functional Annotation

Miranda was used to predict the target genes based on the mRNA transcriptome. In our work, in total, 12,819 unigenes were targeted by 647 miRNAs. In addition, 43 miRNAs were predicted to target more than 100 genes; among these, miR-6914-5p was predicted to target 2,258 genes, which is the largest number observed in this study. Also, we found that 3,688 unigenes were targeted by two or more miRNAs. Furthermore, 15 DEGs targeted by 23 miRNAs were predicted (Table 2).

**TABLE 2:** Predicted significant DEGs were targeted by miRNAs.

**Unigene**	**Length (bp)**	**Expression (F vs. C)**	**Accession number**	**Regulatory miRNAs**
*Eriocheir sinensis* caspase mRNA	2,526	Up	TRINITY_DN67527_c1_g1	esi-miR-6914-5p
*Eriocheir sinensis* retinoid X receptor (RXR) mRNA	12,562	Up	TRINITY_DN71171_c0_g3	esi-miR-2b, esi-miR-6914-5p, esi-miR-7911c-5p
*Eriocheir sinensis* C-type lectin mRNA	1,288	Up	TRINITY_DN70126_c0_g1	esi-let-7e-3p
*Eriocheir sinensis* ecdysteroid receptor (EcR) gene	874	Up	TRINITY_DN60221_c3_g5	esi-miR-6599-5p
*Eriocheir sinensis* Down syndrome cell adhesion molecule (Dscam) gene	555	Up	TRINITY_DN56196_c7_g1	esi-miR-9277
*Eriocheir sinensis* clone CX003 microsatellite sequence	417	Up	TRINITY_DN62484_c3_g1	esi-miR-6914-5p
*Eriocheir sinensis* fatty acid-binding protein 9 (FABP9) gene	936	Up	TRINITY_DN63173_c3_g2	esi-miR-1386
*Eriocheir sinensis* WAP2	882	Up	TRINITY_DN66360_c1_g1	esi-miR-6914-5p
NFX1-type zinc finger-containing protein 1	1,382	Up	TRINITY_DN73356_c3_g1	esi-miR-3574
Uncharacterized protein	2,212	Up	TRINITY_DN66345_c1_g1	esi-miR-224, esi-miR-133d, esi-miR-133
Uncharacterized protein	285	Up	TRINITY_DN66345_c0_g2	esi-miR-133c-3p, esi-miR-133d, esi-miR-133, esi-miR-133, esi-miR-133b, esi-miR-133a, esi-miR-133-3p, esi-miR-133-3p
Uncharacterized protein	1,018	Up	TRINITY_DN73807_c1_g3	esi-miR-133c-3p, esi-miR-133d, esi-miR-133, esi-miR-133, esi-miR-133b, esi-miR-133, esi-miR-133b, esi-miR-133a, esi-miR-133-3p, esi-miR-133-3p
Uncharacterized protein	266	Up	TRINITY_DN62223_c4_g2	esi-miR-423-5p, esi-miR-423
Uncharacterized protein	3,240	Up	TRINITY_DN68868_c0_g1	esi-miR-276a-5p, esi-miR-378b
–	757	Up	TRINITY_DN67560_c0_g4	esi-miR-3574

In addition, the predicted target unigenes of 29 DEMs were shown in the Supplementary Material (S1).

To analyze the functions of the target genes among the different groups, most genes were classified into 44 different GO terms. The terms male meiosis, apical cortex, and ARF guanyl-nucleotide exchange factor activity were the most highly enriched in the biological process, cellular component, and molecular function categories, respectively, between the control and fight groups. In addition, 259 target genes were assigned to 169 KEGG pathways. The top 20 enriched pathways are shown in Figure 5 and include the cAMP signaling pathway, MAPK signaling pathway-fly, protein digestion and absorption, and fatty acid biosynthesis.

## Discussion

In this study investigating agonistic behavior in *E. sinensis*, we reveal the underlying molecular mechanisms through an analysis of miRNA and mRNA expression profiles using a deep sequencing approach. We found 29 miRNAs and 116 unigenes with significant differential expression in the fight group. In the 29 DEMs, we did not find any that was characterized in crustaceans, but the mse-miR-281 (*Manduca sexta*) (Zhang et al., 2012), lgi-miR-750 (*Lottia gigantea*) (Song et al., 2018), sme-miR-315-5p (*Schmidtea mediterranea*) (da Silva et al., 2015), cin-miR-4171-5p (*Ciona intestinalis*), and nve-miR-2025-3p (*Nematostella vectensis*) were characterized in invertebrates. And then, we analyzed the functions of the DEMs and found that they play some key roles in the regulation of animal behaviors. For example, esi-miR-199a-5p as a DEM could induce neuronal apoptosis by targeting the HIF-1α gene and then affect learning and memory behaviors in rats (Yan et al., 2017). Besides, miR-199a-5p was believed to participate in the regulation of glucose metabolism (Wu et al., 2015; Yan et al., 2017; Esteves et al., 2018). Agonistic behavior usually was accompanied by energy consumption, especially glucose metabolism (Briffa and Elwood, 2002). Due to the high conservation of miRNAs (Lewis et al., 2005), we assumed that miR-199a-5p may regulate crabs’ agonistic behavior by mediating neurogenesis and energy metabolism.

Based on the analysis of the mRNA transcripts, we also found some DEGs which could regulate energy metabolism. For example, we observed that the KEGG pathway of the RAS was significantly enriched with DEGs (Figure 5). The RAS, which consists of angiotensinogen, angiotensin-converting enzyme (ACE) isoforms, and angiotensin peptides and their receptors, performs many important functions, including glucose homeostasis (Luther and Brown, 2011), electrolyte balance (Jackson et al., 2018), and features of a neurotransmitter (Phillips et al., 1979) in mammals. In the RAS, angiotensinogen is transformed to form angiotensin (Ang) I under renin action and is then cleaved by ACE to form Ang II. Ang II can induce metabolic effects by acting on cell surface G protein-coupled receptors (White et al., 2019). In fact, the RAS has been shown to mediate glucose metabolism (Gamba et al., 2019). As previously discussed, agonistic behavior is usually accompanied by glucose metabolism in crustaceans. Therefore, there is new evidence suggesting that the RAS may participate in the regulation of agonistic behavior in crabs. Based on the above studies, ACE, which was significantly upregulated in our results, plays a key role in the RAS pathway. A series of studies show that ACE is related to glucose and lipid metabolism (Vaughan et al., 2016; Glover et al., 2019). In addition, ACE modulates behavioral and metabolic responses to diet in *Drosophila melanogaster* (Glover et al., 2019). Moreover, ACE may alter anxiety-like behavior (Wang L.A. et al., 2018) and cognitive ability (Gamba et al., 2019) by mediating the RAS. Therefore, we propose that the ACE gene plays a key role in the RAS pathway to alter energy metabolism, thereby affecting agonistic behavior in *E. sinensis*.

Focusing on the neuroregulation in behavior, research show that let-7d played an important role in regulating the attention deficit hyperactivity disorder; Wu et al. (2010) found that an increased level of let-7d could decrease tyrosine hydroxylase activity by targeting galectin-3 and affect DA metabolism in rats (Wu et al., 2010). A recent study also showed that through a D3R target-mediated mechanism, let-7d improved anxiety and depression disorders (Bahi and Dreyer, 2018). The above evidence suggested that let-7d is involved in the upstream and downstream regulation of DA. According to previous studies, DA is a key neurotransmitter that may affect agonistic behavior in crustaceans. For example, Briffa and Elwood (2002) confirmed that the circulating levels of DA significantly decrease after a fight in *Pagurus bernhardus*, and the same result was found in shore crab (*Carcinus maenas*) (Sneddon et al., 2000). In addition, an injection of DA could improve catechol-*O*-methyltransferase (COMT) gene expression, and COMT might be related to aggressiveness in the swimming crab (*Portunus trituberculatus*) (Zhu et al., 2018). In our previous two studies, we also found that the DA level is significantly decreased during agonistic behavior and suggested that an injection of DA reduced agonistic behavior in *E. sinensis* (Pang et al., 2019a; Yang et al., 2019). In contrast to let-7d, two DEMs, that is, esi-miR-200a and esi-miR-200b, exhibited a significant downregulation in the fight group. However, these DEMs also played important roles in regulating the dopaminergic system (Dulcis et al., 2017; Wu et al., 2018). Wu et al. (2018) proved that miR-200a could influence Parkinson’s disease by mediating the DA2 receptor, which regulates the cAMP–PKA signaling pathway. In addition, miR-200b is associated with methamphetamine (METH) addiction, and METH could induce behavioral changes by changing the DA level in rats (Sim et al., 2017). Thus, the significant change in esi-let-7d, esi-miR-200a, and esi-miR-200b indicated that these factors may influence agonistic behavior in *E. sinensis* by mediating the DA regulation mechanism. However, the different expression levels among esi-miR-200a, esi-miR-200b, and esi-let-7d suggest that these factors may play different roles in the regulation of agonistic behavior.

In our results, we predict that the *E. sinensis* retinoid X receptor (RXR) as a DEG was targeted by five miRNAs (Table 2). In the eyes, retinoic acid (RA) is responsible for phototransduction through binding the G-protein receptors (Uray et al., 2016). The activity of RA can be regulated by RXR, which is important for the proper function of the retina (Janssen et al., 1999). RXR can regulate the development of cone photoreceptors in many animals, and the first step of the phototransduction of vision requires cone photoreceptors (Hennig et al., 2008). To the best of our knowledge, the visual systems are widely used for animal communication, including color discrimination (Chiou et al., 2011), individual recognition (Gherardi et al., 2010), and other animal behaviors (Aquiloni and Gherardi, 2010). Undoubtedly, differences in visual signals can also induce changes in the behavioral responses of crustaceans. For example, mantis shrimp (*Squilla empusa*) displayed varying degrees of agonistic behavior according to vision differences (Wortham-Neal, 2002). In summary, we propose that visual signals were produced when the two crabs met, and then, the signals were conducted into the animals’ CNS by receptors that may contain RXR. Subsequently, the command for agonistic behavior is issued by the CNS. Of course, the behavior may be regulated by complex physiological processes, such as neurotransmission, energy metabolism, and immunoregulation. Based on our results, genes other than those discussed above exhibited significant expression differences. Due to the length of this paper, we only analyzed the DEGs considered to perform considerable functions in the regulation of agonistic behavior. The other DEGs will be further explored in future studies.

Analyzing the predicted target unigene enrichment KEGG pathways (Figure 5), we found that the cAMP signaling pathway is the most enriched. The cAMP is a second messenger that can be activated by neuromodulators and then affect protein kinase A (PKA) activity (Buckley et al., 2016; Momohara et al., 2016). According to reports, the cAMP–PKA signaling pathway was detected to be essential for mediating loser and winner effects in agonistic behavior (Momohara et al., 2016). Our previous studies also showed that an injection of CPT-cAMP into crabs inhibited agonistic behavior in *E. sinensis* (Pang et al., 2019a). Therefore, the results of this study further demonstrate that the cAMP signaling pathway participates in the regulation of agonistic behavior. And the cAMP pathway played an important role in the energy metabolism and the conduction of nerve signals in crustaceans (Pang et al., 2019a; Yang et al., 2019). Also, we found that the MAPK pathway was significantly enriched with unigenes. The MAPK pathway is a three-tiered cascade (MAP3Ks–MAP2Ks–MAPKs), and the MAPK family includes extracellular signal−regulated kinase (ERK), P38, and c−Jun NH2−terminal kinase (JNK) (Malki et al., 2014). Several studies have reported that the MAPK pathway is linked to highly aggressive behavior (Malki et al., 2014; Malki et al., 2016a, b). In MAPK signaling cascades, ERK seems to be mainly associated with aggression (Malki et al., 2014). ERK is a key factor in the MAPK pathway that has been revealed to be involved in energy metabolism (Kuo et al., 2019). In fact, our results further show that the protein digestion and absorption pathway and fatty metabolism pathway are significantly enriched with DEGs. As previously mentioned, energy metabolism may be regulated by ERK or other key factors involved in agonistic behavior. To sum up, this study suggested that the regulation between miRNAs and genes could mediate the energy metabolism and the neurogenesis to regulate the agonistic behavior in *E. sinensis*.

## Conclusion

In this paper, we performed an integrated analysis of miRNA and mRNA expression profiles and found that the miRNAs and mRNAs were involved in the behavior by mediating the progress of neurogenesis and energy metabolism in *E. sinensis*. We propose that DEMs and DEGs, mainly including esi-miR-199a-5p, esi-let-7d, the esi-miR-200 family, RXR, and ACE genes, may regulate agonistic behavior. Additionally, the miRNAs and mRNAs mainly focus on the RAS, cAMP, MAPK, and energy metabolism pathways after a fight. These results reveal the underlying molecular mechanisms of agonistic behavior and could guide researchers in investigating behavioral plasticity in the Chinese mitten crab.

## Data Availability Statement

The datasets generated for this study can be found in the https://dataview.ncbi.nlm.nih.gov/object/PRJNA565886?reviewer=ugafl4uq46l71e8ro649glsvhh.

## Ethics Statement

The animal study was reviewed and approved by the Animal Experiments Ethics Committee of Shanghai Ocean University.

## Author Contributions

The experiments of this study were designed by YP and XY. YS, XS, and JL assisted YP and LH to complete several animal experiments. Results analysis and manuscript writing were made by YP, and then were reviewed and edited by XY. The funding resources came from YC. All authors read and approved the final manuscript.

## Conflict of Interest

The authors declare that the research was conducted in the absence of any commercial or financial relationships that could be construed as a potential conflict of interest.

## Footnotes

1 http://www.kegg.jp/blastkoala/

## References

[B1] Aquiloni L. Gherardi F. (2010). Crayfish females eavesdrop on fighting males and use smell and sight to recognize the identity of the winner. *Anim. l Behav.* 79 265–269. 10.1016/j.anbehav.2009.09.024

[B2] Bahi A. Dreyer J. L. (2018). Lentiviral-mediated let-7d microRNA overexpression induced anxiolytic- and anti-depressant-like behaviors and impaired dopamine D3 receptor expression. *Eur. Neuropsychopharmacol.* 28 1394–1404. 10.1016/j.euroneuro.2018.09.004 30244920

[B3] Bartel D. P. (2004). MicroRNAs: genomics, biogenesis, mechanism, and function. *Cell* 116 281–297. 14744438 10.1016/s0092-8674(04)00045-5

[B4] Betel D. Wilson M. Gabow A. Marks D. S. Sander C. (2008). The microRNA. org resource: targets and expression. *Nucleic Acids Res.* 36 D149–D153. 18158296 10.1093/nar/gkm995 PMC2238905

[B5] Bolger A. M. Lohse M. Usadel B. (2014). Trimmomatic: a flexible trimmer for Illumina sequence data. *Bioinformatics* 30 2114–2120. 10.1093/bioinformatics/btu170 24695404 PMC4103590

[B6] Briffa M. Elwood R. W. (2002). Power of shell-rapping signals influences physiological costs and subsequent decisions during hermit crab fights. *Proc.: Biol. Sci.* 269 2331–2336. 10.1098/rspb.2002.2158 12495500 PMC1691162

[B7] Buckley S. J. Fitzgibbon Q. P. Smith G. G. Ventura T. (2016). In silico prediction of the G-protein coupled receptors expressed during the metamorphic molt of *Sagmariasus verreauxi* (*Crustacea*: *Decapoda*) by mining transcriptomic data: RNA-seq to repertoire. *Gen. Comp. Endocrinol.* 228 111–127. 10.1016/j.ygcen.2016.02.001 26850661

[B8] Cheng Y. X. Wu X. G. Li J. Y. (2018). “Chinese mitten crab culture: current status and recent progress towards sustainable development,” in *Aquaculture in China*, eds Gui J-F Tang Q. Zhongjie L. De Silva S. S. Jiashou L. (Hoboken, NJ: John Wiley & Sons, Limited), 55–69.

[B9] Chiou T. H. Marshall N. J. Caldwell R. L. Cronin T. W. (2011). Changes in light-reflecting properties of signalling appendages alter mate choice behaviour in a stomatopod crustacean *Haptosquilla trispinosa*. *Mar. Freshw. Behav. Physiol.* 44 1–11. 10.1080/10236244.2010.546064

[B10] da Silva W. dos Santos R. A. S. Moraes K. C. M. (2015). Mir-351-5p contributes to the establishment of a pro-inflammatory environment in the H9c2 cell line by repressing PTEN expression. *Mol. Cell. Biochem.* 411 363–371. 10.1007/s11010-015-2598-5 26541756

[B11] Dulcis D. Lippi G. Stark C. J. Do L. H. Berg D. K. Spitzer N. C. (2017). Neurotransmitter switching regulated by miRNAs controls changes in social preference. *Neuron* 95 1319.e5–1333.e5. 28867550 10.1016/j.neuron.2017.08.023 PMC5893310

[B12] Esteves J. V. Yonamine C. Y. Pinto D. C. Gerlinger-Romero F. Enguita F. J. Machado U. F. (2018). Diabetes modulates microRNAs 29b-3p, 29c-3p, 199a-5p and 532-3p expression in muscle: possible role in GLUT4 and HK2 repression. *Front. Endocrinol.* 9:12 10.3389/fendo.2018.00536 PMC6143689 30258406

[B13] Gamba P. Staurenghi E. Testa G. Giannelli S. Sottero B. Leonarduzzi G. (2019). A crosstalk between brain cholesterol oxidation and glucose metabolism in Alzheimer’s disease. *Front. Neurosci.* 13:9 10.3389/fnins.2019.00556 PMC6554318 31213973

[B14] Gherardi F. Cenni F. Parisi G. Aquiloni L. (2010). Visual recognition of conspecifics in the American lobster. Homarus americanus. *Ani. Behav.* 80 713–719. 10.1016/j.anbehav.2010.07.008

[B15] Glover Z. Hodges M. D. Dravecz N. Cameron J. Askwith H. Shirras A. (2019). Loss of angiotensin-converting enzyme-related (ACER) peptidase disrupts behavioural and metabolic responses to diet in *Drosophila melanogaster*. *J. Exp. Biol.* 222:jeb194332 10.1242/jeb.194332 30940674

[B16] Harlıoğlu M. M. Harlıoğlu A. G. Mişe Yonar S. Çakmak Duran T. (2013). Effects of dietary l-tryptophan on the agonistic behavior, growth, and survival of freshwater crayfish *Astacus leptodactylus* Eschscholtz. *Aqu. Int.* 22 733–748. 10.1007/s10499-013-9702-1

[B17] Hennig A. K. Peng G. H. Chen S. M. (2008). Regulation of photoreceptor gene expression by Crx-associated transcription factor network. *Brain Res.* 1192 114–133. 10.1016/j.brainres.2007.06.036 17662965 PMC2266892

[B18] Jackson L. Eldahshan W. Fagan S. C. Ergul A. (2018). Within the brain: the renin angiotensin system. *Int. J. Mol. Sci.* 19 876. 10.3390/ijms19030876 PMC5877737 29543776

[B19] Janssen J. J. M. Kuhlmann E. D. van Vugt A. H. M. Winkens H. J. Janssen B. P. M. Deutman A. F. (1999). Retinoic acid receptors and retinoid X receptors in the mature retina: subtype determination and cellular distribution. *Curr. Eye Res.* 19 338–347. 10.1076/ceyr.19.4.338.5307 10520230

[B20] Jensen K. P. Covault J. Conner T. S. Tennen H. Kranzler H. R. Furneaux H. M. (2009). A common polymorphism in serotonin receptor 1B mRNA moderates regulation by miR-96 and associates with aggressive human behaviors. *Mol. Psychiatry* 14 381–389. 10.1038/mp.2008.15 18283276 PMC3162374

[B21] Kozomara A. Griffiths-Jones S. (2014). miRBase: annotating high confidence microRNAs using deep sequencing data. *Nucleic Acids Res.* 42 D68–D73. 24275495 10.1093/nar/gkt1181 PMC3965103

[B22] Kuo Y.-T. Lin C.-C. Kuo H.-T. Hung J.-H. Liu C.-H. Jassey A. (2019). Identification of baicalin from bofutsushosan and daisaikoto as a potent inducer of glucose uptake and modulator of insulin signaling-associated pathways. *J. Food Drug Anal.* 27 240–248. 10.1016/j.jfda.2018.07.002 30648577 PMC9298638

[B23] Lane B. J. Kick D. R. Wilson D. K. Nair S. S. Schulz D. J. (2018). Dopamine maintains network synchrony via direct modulation of gap junctions in the crustacean cardiac ganglion. *Elife* 7:18. 10.7554/eLife.39368 PMC6199132 30325308

[B24] Laranja J. L. Q. Quinitio E. T. Catacutan M. R. Coloso R. M. (2010). Effects of dietary l-tryptophan on the agonistic behavior, growth and survival of juvenile mud crab *Scylla serrata*. *Aquaculture* 310 84–90. 10.1016/j.aquaculture.2010.09.038

[B25] Lewis B. P. Burge C. B. Bartel D. P. (2005). Conserved seed pairing, often flanked by adenosines, indicates that thousands of human genes are microRNA targets. *Cell* 120 15–20. 10.1016/j.cell.2004.12.035 15652477

[B26] Li X. K. Jin P. (2010). Roles of small regulatory RNAs in determining neuronal identity. *Nat. Rev. Neurosci.* 11 329–338. 10.1038/nrn2739 20354535

[B27] Liu X. Luo B. Y. Feng J. B. Zhou L. X. Ma K. Y. Qiu G. F. (2019). Identification and profiling of microRNAs during gonadal development in the giant freshwater prawn *Macrobrachium rosenbergii*. *Sci. Rep.* 9:2406. 10.1038/s41598-019-38648-x PMC6382778 30787336

[B28] Luther J. M. Brown N. J. (2011). The renin-angiotensin-aldosterone system and glucose homeostasis. *Trends Pharmacol. Sci.* 32 734–739. 21880378 10.1016/j.tips.2011.07.006 PMC3223326

[B29] Malki K. Du Rietz E. Crusio W. E. Pain O. Paya-Cano J. Karadaghi R. L. (2016a). Transcriptome analysis of genes and gene networks involved in aggressive behavior in mouse and zebrafish. *Am. J. Med. Gen. Part B Neuropsychiatr. Gen.* 171 827–838. 10.1002/ajmg.b.32451 27090961

[B30] Malki K. Tosto M. G. Pain O. Sluyter F. Mineur Y. S. Crusio W. E. (2016b). Comparative mRNA analysis of behavioral and genetic mouse models of aggression. *Am. J. Med. Gen. Part B Neuropsychiatr. Gen.* 171B 427–436. 10.1002/ajmg.b.32424 26888158

[B31] Malki K. Pain O. Du Rietz E. Tosto M. G. Paya-Cano J. Sandnabba K. N. (2014). Genes and gene networks implicated in aggression related behaviour. *Neurogenetics* 15 255–266. 10.1007/s10048-014-0417-x 25142712

[B32] Momohara Y. Aonuma H. Nagayama T. (2018). Tyraminergic modulation of agonistic outcomes in crayfish. *J. Comp. Physiol.Neuroethol. Sens. Neural Behav. Physiol.* 204 465–473. 10.1007/s00359-018-1255-3 29488014

[B33] Momohara Y. Minami H. Kanai A. Nagayama T. (2016). Role of cAMP signalling in winner and loser effects in crayfish agonistic encounters. *Eur. J. Neurosci.* 44 1886–1895. 10.1111/ejn.13259 27086724

[B34] Mulloney B. Smarandache-Wellmann C. (2012). Neurobiology of the crustacean swimmeret system. *Prog. Neurobiol.* 96 242–267. 10.1016/j.pneurobio.2012.01.002 22270044 PMC3297416

[B35] Nawrocki E. P. Burge S. W. Alex B. Jennifer D. Eberhardt R. Y. Eddy S. R. (2015). Rfam 12.0: updates to the RNA families database. *Nucleic Acids Res.* 43 D130–D137. 25392425 10.1093/nar/gku1063 PMC4383904

[B36] Pang Y. Y. Song Y. M. Zhang L. Song X. Z. Zhang C. Lv J. H. (2019a). 5-HT2B, 5-HT7, and DA2 receptors mediate the effects of 5-HT and DA on agonistic behavior of the Chinese mitten crab (*Eriocheir sinensis*). *ACS Chem. Neurosci.* 10 4502–4510. 10.1021/acschemneuro.9b00342 31642670

[B37] Pang Y. Y. Zhang C. Xu M. J. Huang G. Y. Cheng Y. X. Yang X. Z. (2019b). The transcriptome sequencing and functional analysis of eyestalk ganglions in Chinese mitten crab (*Eriocheir sinensis*) treated with different photoperiods. *PloS One* 14:e0210414 10.1371/journal.pone.0210414 PMC6333377 30645610

[B38] Phillips M. I. Weyhenmeyer J. Felix D. Ganten D. Hoffman W. E. (1979). Evidence for an endogenous brain renin-angiotensin system. *Federation Proc.* 38 2260–2266. 222621

[B39] Rivetti C. Campos B. Pina B. Raldua D. Kato Y. Watanabe H. (2018). Tryptophan hydroxylase (TRH) loss of function mutations induce growth and behavioral defects in *Daphnia magna*. *Sci. Rep.* 8:1518. 10.1038/s41598-018-19778-0 PMC5784079 29367674

[B40] Romano N. Zeng C. (2016). Cannibalism of decapod crustaceans and implications for their aquaculture: a review of its prevalence, influencing factors, and mitigating methods. *Rev Fish. Sci. Aqu.* 25 42–69. 10.1080/23308249.2016.1221379

[B41] Sim M. S. Soga T. Pandy V. Wu Y. S. Parhar I. S. Mohamed Z. (2017). MicroRNA expression signature of methamphetamine use and addiction in the rat nucleus accumbens. *Metab. Brain Dis.* 32 1767–1783. 10.1007/s11011-017-0061-x 28681200

[B42] Sneddon L. U. Taylor A. C. Huntingford F. A. Watson D. G. (2000). Agonistic behaviour and biogenic amines in shore crabs *Carcinus maenas*. *J. Exp. Biol.* 203 537–545. 10637182 10.1242/jeb.203.3.537

[B43] Song W. Zhu Y. F. Wang L. M. Jiang K. J. Zhang F. Y. Ma C. Y. (2018). Identification and profiling of microRNAs of *Euphausia superba* using Illumina deep sequencing. *J. Oceanol. Limnol.* 36 2278–2287. 10.1007/s00343-019-7229-7

[B44] Thongbuakaew T. Suwansa-ard S. Sretarugsa P. Sobhon P. Cummins S. F. (2019). Identification and characterization of a crustacean female sex hormone in the giant freshwater prawn. Macrobrachium rosenbergii. *Aquaculture* 507 56–68. 10.1016/j.aquaculture.2019.04.002

[B45] Uray I. P. Dmitrovsky E. Brown P. H. (2016). Retinoids and rexinoids in cancer prevention: from laboratory to clinic. *Semin. Oncol.* 43 49–64. 10.1053/j.seminoncol.2015.09.002 26970124 PMC4789177

[B46] Vaughan D. Brogioli M. Maier T. White A. Waldron S. Rittweger J. (2016). The angiotensin converting enzyme insertion/deletion polymorphism modifies exercise-induced muscle metabolism. *PloS One* 11:20 10.1371/journal.pone.0149046 PMC4794249 26982073

[B47] Vomel M. Wegener C. (2008). Neuroarchitecture of aminergic systems in the larval ventral ganglion of *Drosophila melanogaster*. *PloS One* 3:18 10.1371/journal.pone.0001848 PMC2268740 18365004

[B48] Wang L. Xu X. Yang J. Chen L. Liu B. Liu T. (2018). Integrated microRNA and mRNA analysis in the pathogenic filamentous fungus *Trichophyton rubrum*. *BMC Genomics* 19:933 10.1186/s12864-018-5316-3 PMC6295003 30547762

[B49] Wang L. A. de Kloet A. D. Smeltzer M. D. Cahill K. M. Hiller H. Bruce E. B. (2018). Coupling corticotropin-releasing-hormone and angiotensin converting enzyme 2 dampens stress responsiveness in male mice. *Neuropharmacology* 133 85–93. 10.1016/j.neuropharm.2018.01.025 29360543 PMC5858993

[B50] White M. C. Fleeman R. Arnold A. C. (2019). Sex differences in the metabolic effects of the renin-angiotensin system. *Biol. Sex Differ.* 10:31. 10.1186/s13293-019-0247-5 PMC6604144 31262355

[B51] Wortham-Neal J. L. (2002). Intraspecific agonistic interactions of *Squilla empusa* (*Crustacea* : *Stomatopoda*). *Behaviour* 139 463–486. 10.1163/15685390260135961

[B52] Wu C. Lv C. Chen F. Q. Ma X. Y. Shao Y. Wang Q. Y. (2015). The function of miR-199a-5p/Klotho regulating TLR4/NF-kappa B p65/NGAL pathways in rat mesangial cells cultured with high glucose and the mechanism. *Mol. Cell. Endocrinol.* 417 84–93. 10.1016/j.mce.2015.09.024 26419931

[B53] Wu D. M. Wang S. Wen X. Han X. R. Wang Y. J. Shen M. (2018). Inhibition of microRNA-200a upregulates the expression of striatal dopamine receptor D2 to repress apoptosis of striatum via the cAMP/PKA signaling pathway in rats with Parkinson’s disease. *Cell. Physiol. Biochem.* 51 1600–1615. 10.1159/000495649 30497067

[B54] Wu L. H. Zhao Q. L. Zhu X. C. Peng M. Jia C. Y. Wu W. (2010). A novel function of microRNA Let-7d in regulation of galectin-3 expression in attention deficit hyperactivity disorder rat brain. *Brain Pathol.* 20 1042–1054. 10.1111/j.1750-3639.2010.00410.x 20557304 PMC8094722

[B55] Xiaobei Z. Helen L. Robinson M. D. (2014). Robustly detecting differential expression in RNA sequencing data using observation weights. *Nucleic Acids Res.* 42:e91 10.1093/nar/gku310 PMC4066750 24753412

[B56] Yan J. Yu Y. Sun Y. Hu R. Jiang H. (2017). Ketamine induces neuronal apoptosis and cognitive disorder via miR-199a-5p/HIF-1α in neonatal rats. *Mol. Cell. Toxicol.* 13 395–404. 10.1007/s13273-017-0044-3

[B57] Yang X. Z. Pang Y. Y. Huang G. Y. Xu M. J. Zhang C. He L. (2019). The serotonin or dopamine by cyclic adenosine monophosphate-protein kinase A pathway involved in the agonistic behaviour of Chinese mitten crab, *Eriocheir sinensis*. *Physiol. Behav.* 209:112621 10.1016/j.physbeh.2019.112621 31323296

[B58] Zhang X. F. Zheng Y. Jagadeeswaran G. Ren R. Sunkar R. Jiang H. B. (2012). Identification and developmental profiling of conserved and novel microRNAs in *Manduca sexta*. *Insect Biochem. Mol. Biol.* 42 381–395. 10.1016/j.ibmb.2012.01.006 22406339 PMC3340478

[B59] Zhang Y. Yang L. Ling C. Heng W. (2018). HuR facilitates cancer stemness of lung cancer cells via regulating miR-873/CDK3 and miR-125a-3p/CDK3 axis. *Biotechnol. Lett.* 40 623–631. 10.1007/s10529-018-2512-9 29344850

[B60] Zhu F. Fu Y. Y. Mu C. K. Liu L. Li R. H. Song W. W. (2018). Molecular cloning, characterization and effects of catechol-omethyltransferase (comt) mrna and protein on aggressive behavior in the swimming crab *Portunus trituberculatus*. *Aquaculture* 495 693–702. 10.1016/j.aquaculture.2018.06.055

